# Divergent alterations in the skeletal muscle and serum proteome in a rodent model of anorexia nervosa and weight recovery

**DOI:** 10.21203/rs.3.rs-8079717/v1

**Published:** 2025-11-18

**Authors:** Megan Rosa-Caldwell, Pieter Koopmans, L Breithaupt, Towia Libermann, Simon Dillon, Gu Xuesong, Kevin Murach, Ursula Kaiser, Seward Rutkove, Christos Mantzoros

**Affiliations:** University of Arkansas; Brigham and Women’s Hospital; Department of Internal Medicine, Boston VA Healthcare System and Beth Israel Deaconess Medical Center, Harvard Medical School

## Abstract

Anorexia nervosa (AN) is a severe psychiatric disorder characterized by prolonged caloric restriction and significant weight loss, including loss of skeletal muscle. However, physiological changes during AN and following weight restoration are poorly understood, especially skeletal muscle physiology and how it relates to systemic physiology. In this study, we utilized a rodent model of AN and weight gain to investigate proteomic changes across the skeletal muscle and serum and how these proteomic alterations relate to muscle functional outcomes. Using SOMAscan proteomics, we identified extensive alterations in the skeletal muscle proteome during 30 days of simulated AN, with the majority of these changes persisting after weight restoration. Muscle mass and strength were not fully recovered after weight restoration, aligning with sustained dysregulation of proteins related to apoptosis, calcium handling, and satellite cell proliferation in skeletal muscle. By contrast, serum proteomic changes were modest during AN but markedly altered and largely distinct from muscle during recovery. TRAIL (TNF-related apoptosis-inducing ligand) emerged as a potential non-invasive biomarker of muscle health as TRAIL content correlated between muscle and serum as well as muscle functional assessments. Proteomic biomarkers of muscle versus systemic AN are largely unique, underlining the complexity of this illness and the need for novel analytical approaches; however, TRAIL dysregulation may be a conserved feature across tissues and should be further investigated as potential non-invasive biomarker of muscle health and a possible mechanistic target for AN-induced muscle loss.

## Introduction

Anorexia Nervosa (AN) and its associated complications remain a significant public health challenge for clinicians, patients, and their families. Anorexia Nervosa affects ~ 4% of women and 0.5% of men in the United States^1–3^, and these percentages correspond to millions of individuals and billons of dollars in healthcare expenses^4, 5^. AN is also one of the most deadly psychiatric disorders; individuals with AN have a 4.5-fold increased mortality risk compared to those without AN^6^. Despite its prevalence and lethality, treatment options for those with AN are limited and often ineffective, resulting in prolonged disease. This sustained disease resistance necessitates novel research avenues to understand the complex interplay of physiology and psychology in this population.

Along with fat loss, the implications of which largely through leptin have been studied extensively^7, 8^, there is a concurrent loss of skeletal muscle during AN. This skeletal muscle loss has been largely uninvestigated across clinical or basic studies of AN. However, recent evidence demonstrates skeletal muscle loss is a significant predictor of morbidity across multiple pathologies such as cancer, aging, others^9^. It is therefore plausible that low muscle mass during AN may be an under investigated contributor to the overall pathophysiology of the condition and contribute to treatment challenges. Our group’s recent systematic review found ~ 25% lower skeletal muscle mass/strength in those with AN compared to healthy controls, which is not fully recovered after weight restoration (~ 9% lower)^10^. Other recent studies have provided some mechanistic insights for low muscle mass in those with AN. For example, our previous works found altered Leukemia Inhibitory Factor Receptor (LIF-R) in the serum of individuals with AN^11^. LIF-R is a key modulator of cancer-associated muscle wasting^12^. LIF-R is also one of the few proteins that differentiated individuals with AN from healthy controls. Other recent works suggest acute alterations to both protein synthetic signaling during AN, which are only partially restored with weight recovery^13^. Emerging evidence therefore suggests that skeletal muscle physiology may be an important contributor to physiological alterations concurrent with AN. Indeed, the myostatin-activin-follistatin pathway has recently been implicated as a key moderator of loss of muscle functional performance in relative energy deficiency in sport (RED-S)^14^. Yet, to our knowledge, in-depth biological evaluation of the skeletal muscle during extreme caloric deprivation characteristic of AN does not exist.

Currently, most of our biological understanding of physiological alterations during AN are based solely on blood-based biomarkers. While these markers are clearly important and less invasive than alternative approaches, more direct assessment of skeletal muscle alterations are necessary to fully understand the biological implications of skeletal muscle health during AN and following weight restoration. In this study, we leveraged our recently published rodent model of AN and recovery to evaluate proteomic alterations within the skeletal muscle and the serum during AN and following weight restoration. We find broad proteomic alterations to the skeletal muscle that are not resolved following weight restoration. Moreover, we find proteomic changes in the skeletal muscle are unique in the serum, but that conserved biomarkers of systemic and skeletal muscle disruption during AN may exist. Taken together, these data strongly imply physiological and musculoskeletal consequences of AN are longer lasting and more difficult to identify than previously anticipated.

## Methods

### Animals Experiments:

All procedures were approved by the Beth Israel Deaconess Medical Center Institutional Animal Care and Use Committee (AUP: 009–2022). We used our recently refined model of AN and AN recovery, which includes group housing rodents for the duration of simulated AN instead of single housing^15^. These rodents are subsets of previously reported studies from our laboratory^13^. All rats were housed in a temperature and humidity-controlled facility kept at ~ 23°C with a 12:12 hour light:dark cycle. At 8 weeks of age, female Sprague Dawley rats began simulated AN protocol, consisting of ~ 50–60% food restriction. Food consumption was assessed 3 days prior to initiation of simulated AN to obtain a baseline food measurement. AN rats were fed ~ 8:00 AM each day, wherein they were moved to individual cages for feeding and allowed up to 2 hours to consume food. Rats were then returned to group housing until feeding the following day. Simulated AN occurred for 30 days, afterwards one cohort of rats (AN, n = 8) were anesthetized with 2.5% isoflurane and gastrocnemius muscles collected and snap frozen in liquid nitrogen for further analysis. Rats were euthanized while under anesthesia by cardiac puncture. Another cohort of rats was provided food *ad libitum* after simulated AN to mirror weight recovery (AN-R, n = 8). AN-R rats continued recovery interventions for 30 days. Afterwards, rats were anesthetized with isoflurane (~ 3%), and gastrocnemius muscles collected for further analysis. Control (CON) rats consumed food *ad libitum* throughout the protocol and were age-matched to AN-R. All gastrocnemius muscles and serum were collected in the fed state (~ 90 min after feeding) using a standardized amount of food for all groups (~ 3 grams of food).

#### Nerve stimulated force production

Maximal force production of the plantar flexor muscles (encompassing the gastrocnemius) was completed ~ 24 hours prior to euthanasia and completed similar to our previous reports^16^. Briefly, rats were anesthetized with ~ 2.5% isoflurane. Their tibial nerve was then stimulated with electrode needles to plantar flexor muscle contraction. Small twitches (10 Hz) were used to ensure appropriate needle placement; thereafter rats received one maximal stimulation at 200 Hz for 200 ms. Graphs of maximal tetanus were visually inspected to ensure a full tetanic contraction was achieved.

#### Grip Strength

Maximal rear paw grip strength was completed ~ 24 hours prior to euthanasia as we have described^17^. Briefly, rats were gently restrained by a trained investigator and the two hindlimb paws placed on a grip bar attached to a force transducer. The rat was then gently pulled from the transducer until the rat released both paws. The corresponding force was then recorded. The procedure was repeated three times and the maximal measurement was used for analysis.

#### SomaScan

Serum and skeletal muscle tissue underwent SOMAscan discovery proteomics at the Genomics, Proteomics, Bioinformatics and Systems Biology Center at Beth Israel Deaconess Medical Center. Briefly, the SOMAscan platform is aptamer-based technology that utilizes single-stranded DNA chemically modified to enhance protein binding with high specificity to measure > 1,300 proteins^18, 19^. Samples were prepared following manufacturer’s recommended protocols. Data quality control and normalization procedures were followed as per previous publications^20, 21^.

### Statistical and Pathway Analysis:

Phenotypic data were analyzed with SAS (SAS^®^ Studio Release: 3.81, SAS Institute Inc. Cary, NC) with a one-way ANOVA and a Tukey-adjusted post-hoc p < 0.05. Differentially expressed proteins were determined using SomaLogic’s DataDelve^™^ Statistics analytical platform. Three AN samples were found to have significantly divergent proteomic profiles compared to the remainder of the group. Upon further examination of the data, we noted these animals did not lose as much muscle strength or bodyweight compared to other rats within the group. We therefore set those rats aside for subsequent proteomic analysis. Similarly, three CON serum profiles were set aside as outliers due to substantial differences in their proteomic signatures compared to the rest of the group. We analyzed up and down-regulated proteins among three specific comparisons: AN v. CON, AN v. AN-R and CON v. AN-R. To refine which processes are persistently dysregulated, we performed pathway analyses on proteins persistently dysregulated between AN and AN-R with background correction (background: all proteins detected in dataset). No pathways reached significance at adjusted p < 0.05, so we used a non-adjusted p < 0.001 as our significance threshold. After differentially altered proteins were assessed, we used ShinyGO 0.82, an online web browser platform, for enrichment analyses to evaluate altered Gene Ontology (GO) pathways^22, 23^. Correlations were assessed with GraphPad Prism Version 10.2.3. All raw and analyzed data for this project can be found on this project’s Open Science Framework Page (https://osf.io/vhy8f/overview?view_only=e721e9b44fa0499195e4cab54c6615d3)

## Results

### Simulated AN resulted in lower muscle size and strength, which were not fully resolved following weight gain

Simulated AN resulted in significant differences in bodyweight compared to CON (~ 36%, p < 0.001, Table 1); however, with refeeding bodyweight differences were no longer present between AN-R and CON (~ 5%, p = 0.066, Table 1). AN resulted in lower rear paw grip strength (~ 40%) which was not restored in AN-R rats compared to CON (~ 22% lower, p = 0.010, Table 1). Maximal plantar flexion was ~ 25% lower in AN rats compared to CON (p < 0.001, Table 1). With weight recovery, plantar in AN-R was not different from CON (~ 9% lower, p = 0.105, Table 1). Gastrocnemius mass was ~ 32% lower in AN compared to CON (p < 0.001) and with weight recovery, AN-R was still ~ 12% lower compared to CON (p = 0.003, Table 1).

### Simulated AN resulted in large changes to the skeletal muscle proteome, which were not restored following weight gain

**Figure 1:**
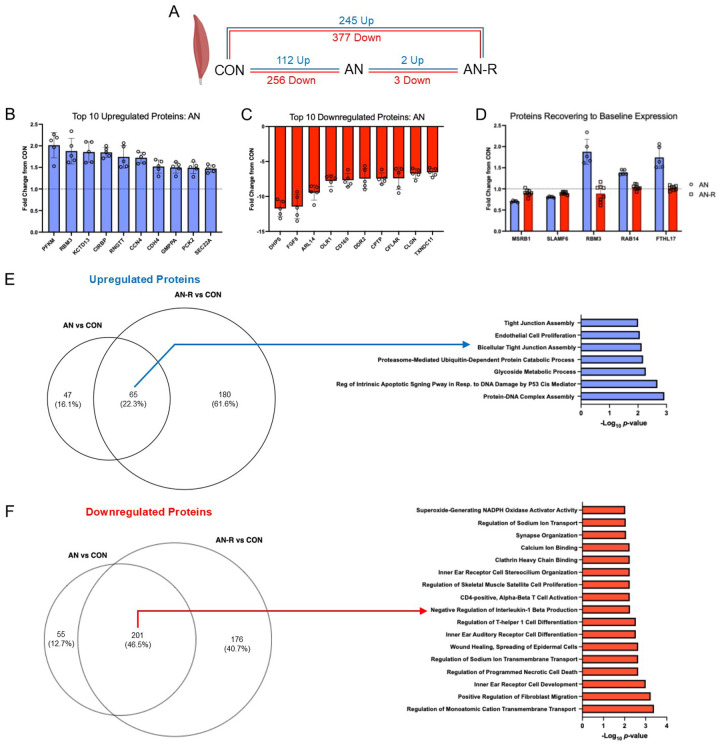
A) Number of differentially abundant proteins between conditions in muscle. Blue font: upregulated. Red front: downregulated. CON: control. AN: Anorexia Nervosa Timepoint. AN-R: Anorexia Nervosa- Recovery Timepoint. B) Top 10 upregulated proteins in AN vs CON. C) Top 10 Downregulated in AN vs CON. D) Fold-changes of the differentially abundant proteins between AN-R and AN through experimental time-course (2 up, 3 down, note: also differentially abundant from AN vs CON). E) Venn diagram comparing number and % of proteins upregulated between AN vs CON and AN-R vs CON. F) Venn diagram comparing number and % proteins downregulated between AN vs CON and AN-R vs CON. All data presented in A-D are adj. p<0.05. E and F are p<0.001. Data presented as mean ± SEM.

We sought to examine alterations to the muscle proteome as a potential driver of muscle mass and functional losses during food-restricted anorexia nervosa (AN) and subsequent recovery with re-feeding (AN-R) in the gastrocnemius muscle. With AN compared to CON timepoint, there were 112 upregulated and 256 downregulated differentially abundant proteins (Fig. 1A). The top 3 differentially abundant upregulated proteins were PFKM, RBM3 and KCTD13 (Fig. 1B). The top 3 abundant downregulated proteins were DHP5, FGF8 and ARL14 (Fig. 1C). The remaining top 10 proteins in each direction are shown in Figs. 1B and 1C. There were only 5 proteins that were altered with AN that returned to baseline with recovery (Fig. 1D), indicating that many of the alterations that occurred during AN were not resolved with weight gain. Instead, we see persistent alteration of the proteome after the recovery phase (AN-R), with 245 upregulated and 377 downregulated proteins compared to CON. We compared the overlap in differentially expressed proteins between the AN and AN-R proteomes and found 65 of 112 (58%) proteins upregulated with AN remained upregulated at the AN-R timepoint relative to CON, and 201 of 256 proteins (78%) downregulated with AN remained downregulated at AN-R timepoint (Fig. 1E and 1F). Some of the top upregulated pathways included protein-DNA complex assembly, apoptotic signaling related to p53, and glycoside metabolic processing (Fig. 1E).

Downregulated pathways related to cation transport, muscle satellite cell proliferation, calcium binding, synapse organization and others (Fig. 1F). Persistent repression of proteins related to the synapse, and binding/transport of cations which are essential for muscle contraction could perhaps explain why volitional muscle strength was not recovered in AN-R rats compared to CON.

### Simulated AN resulted in changes to serum proteome; however, AN-R was more altered compared to AN

**Figure 2:**
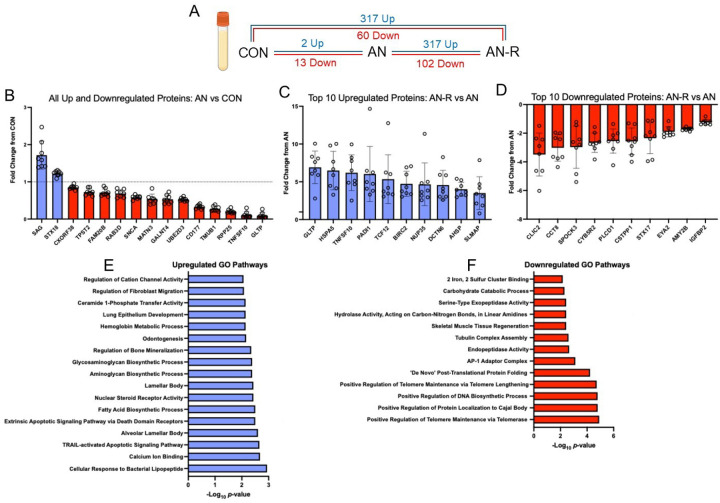
Number of differentially abundant proteins between conditions in serum. Blue font: upregulated. Redfront: downregulated. CON: control. AN: Anorexia Nervosa Timepoint. AN-R: Anorexia Nervosa- Recovery Timepoint. B) Differentially abundant proteins in AN vs CON. C) Top 10 Downregulated in AN-R vs AN. D) Top 10 proteins downregulated in AN-R vs AN. E) Enriched GO terms from proteins upregulated with AN-R vs AN, pathway *p*<0.01. F) Enriched GO terms from proteins downregulated with AN-R vs AN, pathway *p*<0.01.

We next wanted to determine if the serum proteome reflects the muscle proteome during AN and AN-R to identify potential biomarkers of muscle function and size that could be obtained from blood, as muscle biopsies are quite invasive. Only 15 proteins were differentially expressed after AN compared to CON (2 up and 13 down, Fig. 2A). No proteins were up or downregulated in muscle and serum simultaneously at any time point (Supplement A and B). Only one protein, GLTP, a glycolipid transfer protein, was upregulated in muscle while downregulated in serum.

Most changes in the serum proteome occurred with refeeding, with 317 and 102 upregulated and downregulated proteins, respectively, in AN-R versus AN (Fig. 2A, 2C, 2D). We also performed pathway analyses on those proteins (p < 0.001). Upregulated GO terms relate to calcium ion binding and TRAIL (TNF-related apoptosis-induced ligand)-activated apoptotic signaling, apoptotic signaling (Fig. 2F). Downregulated GO terms related to telomere maintenance, Cajal body localization, and endopeptidase activity and others including skeletal muscle tissue regeneration (Fig. 2F)

#### Several proteins are correlated between the skeletal muscle and serum across different experimental timepoints

**Figure 3:**
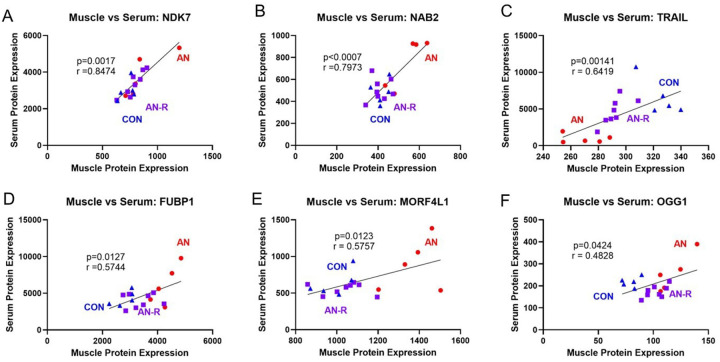
A-F) Animal-matched correlation between muscle vs serum protein expression.

While general patterns between the muscle and serum proteome were not present, we next sought to evaluate if any individual proteins showed convergent patterns between muscle and serum samples. We used animal-matched normalized proteomic values for each sample across timepoints to calculate correlations between muscle and serum. Once significant correlations were identified, individual proteins were further evaluated to assess if correlations were being driven by outliers. We found six proteins that had a significant correlation between muscle and serum (Fig. 3A–F). Of those six, NDK7 had the strongest correlation between muscle and serum (r = 0.847, p = 0.0017, Fig. 3A), followed by NAB2 (r = 0.797, p = 0.0007, Fig. 3B), and TRAIL (TNFSF10, r = 0.642, p = 0.0014, Fig. 3C).

#### Of proteins that correlate between biological samples, several also correlated with muscle function

**Figure 4:**
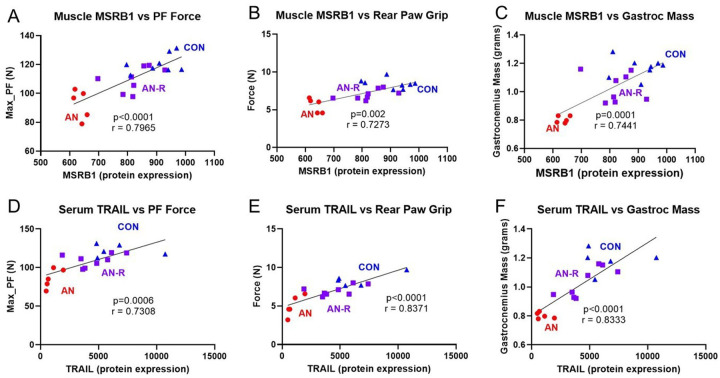
Correlation between muscle MSRB1 protein expression and A) maximal plantar flexor strength. B) rear paw grip, and C) gastrocnemius mass. Correlation between serum TRAIL protein expression and D) maximal plantarflexor strength, E) rear paw grip strength, and F) gastrocnemius mass

We conducted correlations on a few key proteins in muscle and serum in relation to functional outcomes. MSRB1 (methionine sulfoxide reductase B1) was one of the few proteins that was altered with AN and restored in AN-R. Moreover, previous work suggests selenium metabolism is important moderator for disease severity during AN^24^ and may be a key driver of muscle health^25^. Therefore, we first correlated muscle MSRB1 to key metrics of muscle health and performance. Overall MSRB1 had strong correlations with maximal plantar flexion (r = 0.7965, p < 0.0001), rear paw grip strength (r = 0.7273, p = 0.002), muscle fiber cross-sectional area (r = 0.5902, p = 0.0048), and gastrocnemius mass (r = 0.7441, p = 0.0001, Fig. 4A–4D), overall implying some of the proteomic signatures from our rats corresponded to functional outcomes.

Given the previous correlations between serum and muscle proteins for NDK7 and NAB2, we next evaluated if the serum content of these proteins correlated to functional outcomes. Overall, neither protein strongly correlated with any functional parameters (Supplementary Fig. 2), suggesting serum content of these proteins does not appear related to muscle function. However, TRAIL serum content did correlate to muscle functional outcomes including maximal plantar flexion (r = 0.7308, p = 0.0006), rear paw grip strength (r = 0.8371, p < 0.0001), muscle fiber cross-sectional area (r = 0.6876, p = 0.0016), and gastrocnemius mass (r = 0.8333, p < 0.0001, Fig. 4E–4H). Together, TRAIL was the only protein that correlated between muscle and serum and correlated with functional parameters of muscle strength and size.

## Discussion

In this study we utilized a novel proteomic technique to evaluate the proteomic signature of both skeletal muscle and serum in a rodent model of AN and subsequent weight recovery. We found significant alterations to the skeletal muscle proteome during AN that are not resolved with weight recovery. These broad proteomic perturbations mirror phenotypic alterations within the skeletal muscle, with sustained alterations to muscle function despite weight gain. However, within the serum of these same animals, we find relatively minor proteomic changes in AN rats and instead find dramatic alterations in the proteomic signature of the AN-R rats compared to healthy controls. Moreover, the broad proteomic changes within the serum are not generally reflected in the skeletal muscle proteomic changes. In aggregate, these data demonstrate the profound alterations to physiology during AN and suggest prolonged physiological alterations due to AN.

One of the initial goals of this study was to evaluate overlap of serum and skeletal muscle proteomic signatures in our model, potentially allowing for a less invasive and faster (compared to maximal muscle testing) method to evaluate skeletal muscle health. Broadly, we find the proteomic signatures of serum and muscle did not overlap. Specifically, within skeletal muscle we note large changes to the proteome during AN, many of which are not resolved with weight recovery. Contrastingly, within the serum, we find relatively modest changes in the proteome during AN compared to CON, yet with weight recovery (AN-R) there were large alterations compared to either AN or CON. There are a few reasons the skeletal muscle proteome may not fully reflect circulating proteome. First, the serum proteome reflects paracrine activity of the skeletal muscle, but also of all other organs within the body. Given that AN is a whole-body pathology, it is likely other tissues (liver, fat, etc.) are also experiencing profound proteomic modifications. The combination of all of these tissue-specific proteomic changes and corresponding alterations to paracrine activity of these tissues would result in systemic proteomic changes that do not directly mirror tissue-specific proteomic changes. Additionally, the skeletal muscle proteomic changes may reflect alterations to tissue specific biology or changes to the paracrine activity of the skeletal muscle, at present we cannot directly evaluate which transformations within the skeletal muscle tissue are directly altering myokine release from the skeletal muscle and this warrants further investigation. Regardless, our findings suggest proteomic alterations to skeletal muscle and blood are unique during AN and following weight restoration, implying more nuanced or novel techniques will likely be necessary to fully understand the biological ramifications of AN and to develop possible future biomarkers of muscle health that do not require an invasive muscle biopsy or maximal muscle strength assessment.

Our data seemingly contrasts other proteomic studies evaluating the serum proteomic alterations during the low weight phase of AN. These differences may in part be due to controlling for multiple comparisons with the SomaScan platform that evaluates significantly more proteins compared to other proteomic platforms as well as the low number of animals per group. However, prior studies in individuals with AN have tended to suggest inflammatory/cytokine signaling and complement may be key pathways in the etiology of AN^11, 26–28^. Moreover, a recent study evaluating proteomic alterations during acute starvation found similar signaling pathways such as inflammation and complement signaling^29^, suggesting this may be an important physiological alteration that is conserved during severe energy restriction regardless of psychological status. Within our data sets, we also do note some signaling changes that may also support this mechanism such as TRAIL cytokine signaling within the serum and muscle (discussed further below) as well as IL-1Beta and T-helper cell differentiation within the skeletal muscle. However, our data strongly suggests, broad proteomic changes within the skeletal muscle are not reflected in the serum either during acute starvation or during weight restoration. These findings provide context for previous studies and future studies on the reliability of correlating serum findings with skeletal muscle health and further highlight the need for new technologies to non-invasively assess muscle health.

Within the skeletal muscle, many of the top up-regulated and down-regulated proteins during AN are relatively novel in the skeletal muscle literature. These new discoveries may open new avenues to evaluate the relative roles of these proteins in skeletal muscle biology and how they affect systemic health. For example, PFKM is known to be the muscle-specific isoform of PFK, a key glycolytic enzyme. Given limited glucose availability is inherent to AN, the greater abundance of PFKM is a bit paradoxical; however, this specific isoform of PFK may have hitherto unknow roles in muscle biology. Additionally, KCTD13 has previously been reported as a possible genetic contributor to the development of neuropsychiatric diseases^30^. Genetic deletion of KCTD13 inhibited DNA synthesis and proliferation in human-induced pluripotent stem cells^30^. RBM3 is the only top differentially expressed proteins that has previously been reported in skeletal muscle literature. Specifically, the protein is known to be induced in cold environments such as hibernating mammals^31^. More recently, mechanistic studies of RBM3 have demonstrated it is sufficient to improve mitochondrial metabolism and myoblast differentiation^32^. Rodent studies have found RBM3 is elevated during physiological caloric restriction during aging, which is thought to contribute to caloric restriction’s longevity effects^33^. Since there was greater RBM3 content in the present study, it is difficult to fully interpret its role in skeletal muscle physiology and the nuances of RBM3 in relation to pathological (ie starvation/AN) vs. physiological caloric restriction is warranted.

DHPS, FGF8, and ARL14 were the top differentially abundant down-regulated proteins in AN skeletal muscle compared to CON. DHPS is a key protein necessary for activation of eIF5A, which appears necessary for satellite cell activation and presumably muscle growth^34–36^. Moreover, loss of DHPS in zebrafish resulted in significant defects in pancreatic growth, accompanied by changes in gene expression related to mRNA translation, neurogenesis, and stress pathways^37^. Similarly, FGF8 is known to facilitate myogenesis and prevent fat accumulation within skeletal muscle^38^. ARL14 has only been reported within the context of cancer^39^, so its role in skeletal muscle biology remains unclear. In aggregate, considering the top down-regulated proteins are related to muscle growth and remain depressed during AN-R, these results strongly suggest muscle growth pathways are severely altered during AN and simple weight recovery (with duration matching length of starvation) is not sufficient to restore these pathways.

Broadly, the overall patterns in the proteome of the skeletal muscle compared to serum did not have significant overlap, suggesting that broadly serum protein markers are likely not viable methods to estimate muscle health in humans. However, three proteins broadly have values that correlated within the serum and the skeletal muscle, NDK7, NAB2, and TRAIL. While NDK7 and NAB2 overall had strong correlations within the serum and skeletal muscle (r = 0.847 and r = 0.798 respectively), they did not generally correlate with either muscle mass or muscle function outcomes (r range = 0.328–0.541). Suggesting neither of those proteins are necessarily indicative of muscle health per se.

TRAIL content in the skeletal muscle and serum correlated (r = 0.641) and TRAIL content within the serum correlated strongly with muscle size and functional outcomes (range = 0.688–0.837). Functionally, TRAIL is a cytokine within the TNF family of cytokines^40^. Within skeletal muscle, the exact role for TRAIL within skeletal muscle health is not fully clear, with some studies finding TRAIL to negatively regulate muscle differentiation^41^, whereas others have indicated TRAIL is an important moderator of muscle differentiation and Akt signaling^42^. Given the known phenotypic alterations to muscle health in this particular model, the latter explanation of TRAIL activity seems likely; however, more mechanistic studies are likely necessary to fully understand the relevance of TRAIL to muscle biology and subsequent health. Regardless, these correlational data suggest serum TRAIL content may be a viable candidate for estimating muscle health during AN and subsequent recovery in human patients. Previous works in patients with AN have also found lower serum TRAIL content^11, 28^, overall providing additional clinical robustness to our current findings. Therefore, TRAIL content may be a viable candidate to non-invasively estimate muscle health in patients with AN or recovering from AN and additional studies to directly assess this hypothesis in humans are warranted.

There are a few limitations of this study that should be acknowledged. We are likely not fully recapitulating the nuances of a complex psychological disorder such as AN with our rodent model. However, as some of our results in rats appear to replicate some results found in humans, our rodent model appears to at least mimic the physiological responses to severe caloric restriction inherent to AN. Additionally, for this study we utilized a different proteomics technology (SomaScan) compared to other previously reported proteomic based analysis (O-link or GC-MS based platforms). These differences between methods can make direct comparisons between studies difficult. For example, due to the broad range of the SomaScan platform (> 7000 proteins identified) and the mathematical need to correct for multiple comparisons, our analysis may have omitted some proteins that other analysis with smaller protein identification ranges may have identified as significant. Moreover, we cannot account for possible differences in the serum or skeletal muscle proteome based on age. Our CON and AN-R rats are four weeks older than our AN rats in order to age-match these two groups. Therefore, it is possible that some differences may be due to maturation of the rats during the course of the study. Similarly, our results reflect a duration of recovery matching the duration of simulated AN. We cannot determine if proteomic changes are persistent in perpetuity or if these proteomic changes would be resolved given additional recovery time. Finally, a proteomics analysis of the selected tissues only reflects the protein content within that specific tissue and does not indicate what cell types these proteins are being synthesized from (multiple tissues can contribute to the proteomic signature in the serum and there are multiple different cell types within skeletal muscle). These limitations can be addressed in future studies using more targeted proteomic or transcriptomic approaches, such as single-fiber approaches or single-cell RNA sequencing.

In this study we evaluated the proteome of skeletal muscle and serum from a rodent model of AN and subsequent weight recovery. We find the skeletal muscle proteome is largely altered during AN and remains altered during weight restoration. Moreover, the serum proteome is largely altered during weight restoration. These results appear to suggest physiological responses to AN and weight recovery are longer-lasting than previously anticipated. Moreover, we find changes to the skeletal muscle and serum proteome do not broadly overlap, with a few exceptions. Specifically, TRAIL content appears to correlate across tissues and functional outcomes, suggesting assessment of this protein may serve as a viable biomarker in the future and/or may be a possible physiological moderator of muscle derangements during AN. Future research should seek to further clarify the relationship between TRAIL (circulating and muscle specific) and muscle functional outcomes in human patients with AN. If the results from our current investigation are consistent in humans, TRAIL content may offer a novel tool to evaluate physiological recovery from AN that is independent of psychological assessments.

## Supplementary Material

Supplementary Files

This is a list of supplementary files associated with this preprint. Click to download.

- SupplementFig1.jpg
- SupplementFig2.jpg

## Figures and Tables

**Table 1:** 

	CON	AN	AN-R
Bodyweight (g)	234.13 ±4.79^a^	148.80 ± 1.70^b^	221.18 ± 4.28^a^
Rear Paw Grip (N)	8.44 ± 0.22^a^	5.12 ± 0.43^b^	7.0167 ± 0.23^c^
Maximal Plantar Flexion (mN)	120.54 ±2.30^a^	90.35 ± 4.79^b^	109.87 ± 2.97^a^
Gastrocnemius Mass (g)	1.170 ±0.0247^a^	0.7919 ± 0.0123^b^	1.0319 ± 0.0361^c^

Phenotypic data of bodyweight and muscle outcome variables. Data are depicted as Mean + SEM. Different letters represent statistically different at Tukey adjusted p < 0.05. CON = control, AN = anorexia nervosa, AN-R = anorexia nervosa + recovery. N = 8/group

## Data Availability

Raw data and associated statistical code are available at our Open Science Framework page for this project at: https://osf.io/vhy8f/overview?view_only=e721e9b44fa0499195e4cab54c6615d3
